# Royal College of Surgeons in London

**Published:** 1841-10

**Authors:** 


### Royal College of Surgeons in London.

[It appears from the following circular which has been transmitted to us for insertion, that the College of Surgeons is at length arousing from its literary lethargy. When will it open its eyes to its more vital interests, and become in fact as well as in name the College of English Surgeons? Were it not that the history of every country exhibits the fruits, or rather no-fruits, of the *vis inertiæ* of non-representative Corporations of every sort, we should be extremely surprised that a body of learned and enlightened men, like the Council of the London College of Surgeons, should, year after year and for many years, be content to see themselves—or, at least, to be seen—in their collegiate capacity, lagging so far in the rear of improvement. To us and to all men of liberal views not within such mesmeric influence, it seems truly marvellous that the simple truth, approved by the plainest reason and demonstrated by all experience, has not yet forced itself upon their minds—that weak, powerless, and without influence as they know and feel they are in their exclusiveness and isolation, they would be strong and powerful and influential in the fair association, union, and communion of their members. We have heard much, of late, of projects being on foot to accomplish so desirable a change; we know such a change cannot be very long delayed; and we are willing to see in the present feeble glimmer, a promise and harbinger of the day that is at hand. We are the more led to indulge this hope from our knowledge of the great talents, enlightened views, generous sentiments, and energetic character of the present President. We cannot but think that if Mr. Guthrie seriously contemplates the reconstitution of the College on more liberal principles, he has sufficient influence with his colleagues to bring it about: of this, at least, we are certain, that a finer field than that which he now occupies can never present itself for the exercise of the noblest ambition. To the earliest champion of such a cause, even failure will be greater glory than the success which crowns the final effort,—

Victrix caussa Deis placuit sed victa Catoni.

Were the College what it ought to be, the encourager, fosterer, protector,—the true, equal, loving and beloved *alma mater* of all the well-educated surgeons of England, what a different series of Transactions might we expect from that which is now commencing!]

"The Council proposing to publish, in the course of the ensuing year, a volume, to be entitled 'Transactions of the Royal College of Surgeons in London,' invite, from the Members of the College and other scientific persons, communications relating to the improvement of anatomical and surgical Science. The subjects proposed to be included in this Publication are specified in the following extract from the Ordinances of the College: 'The Transactions shall consist of Original Communications on Surgical subjects; Collegial and Jacksonian Prize Dissertations, deemed of sufficient originality and merit; Original Memoirs on Human Anatomy; Original Memoirs on Comparative Anatomy; Anatomical Monographs of rare Animals, dissected in the Museum of the College; Explanations of, and Commentaries on, important Preparations in the Museum, with illustrative Plates; Statistical Reports from Hospitals.'

"It is requested that Papers intended for publication in this volume may be transmitted to the President at the College, on or before the 1st of May, 1842.

"Edmond Belfour, *Secretary*."

